# Integrating oral health into planetary health governance: a framework for resilient and equitable oral healthcare

**DOI:** 10.3389/fdmed.2026.1962649

**Published:** 2026-09-29

**Authors:** Arish Naresh, Roisin McGrath, Easter Joury, Silvana Bettiol, Sheilla Funubo, Anishma Ram, Zaeni Dahlan, Penioni Ravunawa, Peter Iroga, Frank Kade, Najib Baig, Leenu Maimanuku, Diane Pevreal, Lesley Naidoo, William Carlson-Jones, Tan Minh Nguyen, Zac Morse

**Affiliations:** 1 Department of Community Oral Health Service, Health New Zealand Tairāwhiti, Gisborne, New Zealand; 2 College of Health and Medicine, University of Tasmania, Hobart, TAS, Australia; 3 School of Health Sciences, Solomon Islands National University, Honiara, Solomon Islands; 4 Master of Applied Science in Dental Therapy Program, Health Polytechnic Semarang, Semarang, Indonesia; 5 Melbourne Dental School, The University of Melbourne, Melbourne, VIC, Australia; 6 Oral Health Victoria, Melbourne, VIC, Australia; 7 Division of Dentistry, University of Manchester, Manchester, United Kingdom; 8 Public Health and Wellbeing Directorate, London Borough of Sutton, London, United Kingdom; 9 Pasifika Dental Association, Auckland, New Zealand; 10 Department of Policy, The Indonesian Health Council, Jakarta, Indonesia; 11 Ministry of Health and Medical Services, Suva, Fiji Islands; 12 Fiji Oral Health Association, Suva, Fiji Islands; 13 School of Dentistry and Oral Health, College of Medicine, Nursing and Health Sciences, Fiji National University, Suva, Fiji Islands; 14 School of Dentistry, University of KwaZulu-Natal, Durban, South Africa; 15 Dental Therapy Federation of Africa, Durban, South Africa; 16 Sydney Dental School, Faculty of Medicine and Health, University of Sydney, Sydney, NSW, Australia; 17 School of Dentistry, Faculty of Health, Medicine and Behavioural Sciences, University of Queensland, Brisbane, QLD, Australia; 18 Deakin Health Economics, School of Health and Social Development, Institute for Health Transformation, Deakin University, Melbourne, VIC, Australia; 19 Health Economics Group, School of Public Health and Preventive Medicine, Monash University, Melbourne, VIC, Australia; 20 College of Dentistry, American University of Iraq – Baghdad, Baghdad, Iraq

**Keywords:** climate resilience, disaster preparedness, health equity, oral health policy, planetary boundaries, sustainable dentistry

## Abstract

Oral health is largely absent from climate-adaptation and planetary-health governance, even though oral health and oral healthcare depend on supplies of clean water, stable electricity, secure supply chains, and a retained workforce, which climate change is destabilizing. In this Perspective, we argue that this omission is a strategic failure for health security, and draw on the contrasting experiences of seven climate-exposed settings (Australia, Fiji, Indonesia, New Zealand, the Solomon Islands, South Africa, and the United Kingdom) to show how environmental stressors disrupt progress in promoting oral health, thereby deepening oral disease inequities. Across these settings, extreme weather damages clinics, interrupts water-dependent processes such as instrument sterilization and community water fluoridation, undermines workforce retention in the places least able to absorb staff losses, and falls hardest on populations already underserved. We propose a four-part framework that recognizes the need to embed oral health within climate-critical infrastructure, develop region-specific vulnerability assessments, protect the workforce in climate-exposed regions, and establish adaptive financing. These recommendations promote environmental sustainability through prevention-oriented oral health policy and the implementation of reduced carbon-emitting oral healthcare service delivery models.

## 1 Introduction

Oral diseases affect an estimated 3.7 billion people, making them among the most prevalent human health conditions (1–3). Yet, oral health is almost entirely absent from planetary-health governance and climate-adaptation frameworks (4). The World Health Organization (WHO) has placed universal health coverage and health security at the center of its agenda, and oral health is increasingly recognized within the former (2, 3); even so, it is rarely written into disaster-resilience and emergency-preparedness planning as part of health security (5, 6).

Oral disease is generally not self-limiting; it can produce acute infection, pain, sepsis, nutritional compromise, and costly emergency-department presentations (7). When timely oral healthcare access fails, that burden does not disappear but migrates to hospitals at precisely the moment they are least capable of absorbing it. Continuity of oral healthcare, including service delivery models in non-dental settings, is therefore a component of health security in its own right; a point that becomes critical as climate-intensified extreme weather compromises dental infrastructure and widens inequities in the regions least equipped to respond (4, 8, 9). The interruption of routine care during the coronavirus disease 2019 (COVID-19) pandemic prompted the first serious international debate on what constitutes essential oral healthcare and who should deliver it (10).

The planetary-boundaries concept situates human health within the ecological limits that climate change is now testing (11). Oral healthcare delivered in conventional fixed and mobile dental clinics is particularly exposed, sitting at the confluence of four dependencies: (i) clean water for instrument sterilization, handpiece cooling and irrigation, and community water fluoridation; (ii) reliable electricity for autoclaves, suction, and digital workflows; (iii) secure supply chains; and (iv) a workforce able to serve dispersed populations. Drought and water restrictions can threaten a population-level preventive measure such as community water fluoridation (12), while interrupting water supplies for clinical care. Floods inundate and contaminate clinics while exacerbating post-disaster trauma and infection (9). Heat degrades temperature-sensitive materials (6) and, during extremes, threatens power supply (9). Global warming intensifies air pollution, notably ground-level ozone, compounding respiratory and oral morbidity (13, 14). Despite this, oral health is rarely connected systematically to climate adaptation, whether in national policy instruments or in the World Health Organization (WHO) Global Strategy and Action Plan on Oral Health 2023–2030 (2, 15). The occasional appearance in emergency planning is framed around disaster relief rather than climate adaptation (16, 17).

This Perspective, developed by clinicians and researchers working across several climate-exposed countries, advances a single argument: the exclusion of oral health from planetary-health governance is a structural failure of health security, and one that is remediable. Though not designed to be internationally representative, we draw on contrasting selected regional experiences spanning country income levels (18), geographies, and hazard profiles to provide specific examples why the disconnect persists. We propose a four-part framework bridging climate adaptation with environmental sustainability to promote oral health. Our aim is not a primary empirical study but a synthesis of perspectives to inform policy, practice, and transdisciplinary research.

## 2 Oral health within planetary boundaries: a cross-regional view

Climate stressors act on oral health and oral healthcare delivery through a recognizable set of pathways, but the dominant pathway differs sharply across settings (Table 1). The contrast from the seven country examples suggests that vulnerability depends less on geography or income than on the interaction of hazard exposure, infrastructure resilience, and pre-existing disadvantage.

**Table 1:** Dominant climate hazards and principal oral health vulnerabilities across the seven settings discussed, with illustrative adaptation priorities.

Country	Country income	Dominant climate hazards	Principal oral health impact pathways	Illustrative adaptation priorities
Fiji	Upper-middle income	Cyclones; saltwater intrusion; rainfall variability	Damage to coastal clinics; loss of freshwater for sterilization and oral hygiene	Cyclone-resistant clinics; water contingency
Solomon Islands	Lower-middle income	Sea-level rise; drought; cyclones	Groundwater salinization; interrupted hygiene; outer-island isolation	Protected water supply; cyclone-resistant clinics; resilient supply chains
Indonesia	Upper-middle income	Flooding; heat; peatland-fire haze	Facility and WASH disruption (urban); dry-season water scarcity (rural); air-quality burden	Flood-resilient facilities; water contingency; air-quality protocols
South Africa	Upper-middle income	Drought; flooding; heat	Water restriction; damage to rural facilities; HIV-related oral disease	Water contingency and rural facility resilience; equity-focused financing
Australia	High income	Bushfire; heat; drought; flooding; cyclones (northern regions)	Power loss and water scarcity in rural and remote clinics; equipment heat stress; road closure and community isolation interrupting outreach services	Backup power and clinic cooling; flood-resilient access and outreach planning; subnational vulnerability assessment
New Zealand	High income	Flooding; cyclones	Practice closure; care reduced to emergency pain relief	Integration with disaster response; continuity and referral pathways
United Kingdom	High income	Heatwaves; flooding	Cooling-related equipment failures; flood-related facility closures	Clinic cooling; flood protection

In Fiji and the Solomon Islands, water-dependent oral health is acutely exposed to changing rainfall, drought, saltwater intrusion, and intensifying cyclones (19, 20). Cyclones damage coastal-based dental clinics and can sever care for extended periods. Groundwater contamination and dry spells compromise freshwater required for oral healthcare and home oral hygiene. Sea-level rise threatens shallow freshwater that many outer-island communities depend on, with modeling for atolls projecting that wave-driven overwash will render groundwater non-potable around mid-century under high carbon-emission scenarios (21). Dispersed geography compounds this: when dental clinics are inoperable or a supply route fails, the nearest alternative may require a boat journey, and backup capacity is disproportionately costly for small populations (20). Climate disruption to clinic-based care in these states is therefore inseparable from water security, logistics, and ultimately sovereignty (22).

Indonesia illustrates a stark urban–rural divide. In Jakarta, recurrent flooding leaves health facilities and their water and sanitation systems highly exposed (23). Remote archipelagic communities are far less able to absorb such shocks and face compounding dry-season hazards, water scarcity, and peatland-fire haze, whose 2015 crisis was estimated to have caused some 90,000 premature deaths (24). Repeated damage to health facilities accumulates faster than facilities can be restored. The consequences on oral health remain under-examined, and Indonesia's climate–health adaptation planning currently does not visibly extend to oral healthcare.

Australia and New Zealand show that high national income does not confer immunity, although it shapes the form vulnerability takes. Australia experiences weather extremes across the continent, such as prolonged drought, catastrophic bushfires, severe floods, cyclones, and intense heatwaves, amplifying risks to communities, infrastructure, and healthcare delivery (25). Lengthening bushfire seasons disrupt rural and remote services, cutting power to pharmacies and medical centers, forcing the relocation of five regional New South Wales hospitals, and leaving unburnt facilities almost non-functional due to water scarcity and heat (26). Flooding isolates communities when roads are impassable. Rural and remote dental clinics share this exposure, while metropolitan centers are less affected. Within Australia, as across the seven settings, vulnerability depends on infrastructure resilience and pre-existing disadvantage rather than national income. Oral healthcare interruptions remain unresolved in rural and remote areas, particularly for Aboriginal and Torres Strait Islander communities in Western Australia, the Northern Territory, and Queensland, where services are fragmented, workforce turnover is high, and volunteer and outreach models carry a disproportionate share of care (27, 28). Oral health has yet to enter Australia's climate-adaptation planning systematically.

In New Zealand, Cyclone Gabrielle's 2023 flooding closed dental and primary-care services across the worst-hit districts, with some reduced to emergency pain relief only (29), yet oral health remains siloed from disaster-response frameworks. Coordination failures hit rural populations and Indigenous Māori communities hardest (30), underlining that climate adaptation in oral healthcare cannot be separated from equity or the colonial histories that continue to shape who reaches care (8, 31, 32). It is clear that both Australia and New Zealand, as high-income countries, do not confer immunity to climate change.

The United Kingdom has similar vulnerabilities. During the June 2026 heatwave, extreme temperatures and humidity increased demand on health services and exposed weaknesses in aging infrastructure across several National Health Service (NHS) trusts: cooling failures produced excessive temperatures in clinical areas, creating patient-safety risks, and rendered some diagnostic facilities temporarily inoperable (33). At least 67 flood-related closures at NHS sites have been recorded since 2021 (34). Dental clinics share these dependencies on cooling, power, and flood resilience, so vulnerabilities are likely wherever facilities were designed for a climate that no longer exists (6). In a system already struggling with constrained access to NHS dentistry (35), even short interruptions increase dental waiting times, disrupt continuity, and shift demand towards emergency services.

South Africa exemplifies how climate shocks compound entrenched oral healthcare inequity. Drought-driven restrictions limit the clean water on which clinical care and basic oral hygiene depend (4); flooding has damaged health facilities serving rural communities (36); and extreme weather disrupts care for people living with human immunodeficiency virus (HIV) (37), in a population already reporting substantial unmet oral health need (38). Climate exposure interacting with poverty, a high communicable-disease burden, and fragmented services produces disruption that is more severe and persistent than in better-resourced settings, falling hardest on those with the fewest alternatives.

A common climate change challenge emerges. Promoting oral health by relying on oral healthcare systems through dental clinics is bound within ecological limits: freshwater scarcity, extreme weather, and ecosystem stress weaken conventional service delivery models, while the populations least able to adapt (rural, remote, Indigenous, low-income, and chronically ill) bear the greatest cost (11, 31). Three features recur. Water is the limiting factor for clinical care, population-level prevention, and daily oral hygiene. When supply fails, all three fail together. After a disaster, oral health services recover slowly and largely unnoticed (39), because rebuilding funds flow to hospitals rather than dental clinics. And the recovery gap is consistent: those with private, urban, or insured access recover fastest. The insights from seven countries suggest that solutions to climate change resilience for oral health require a shared governance framework.

## 3 Why oral health is missing from climate change resilience governance

Why, then, does oral health remain almost invisible in climate change resilience policy? Reviews of national climate-and-health profiles and adaptation plans consistently document heat stress, vector-borne disease, and threats to health-system infrastructure, but oral health and oral healthcare are seldom protected, and relevant indicators are largely absent from monitoring frameworks (40) and the wider climate–health literature (41). It is also broadly assumed that dental professionals alone are responsible for oral health, constraining how, by whom, and where essential oral healthcare is delivered. Disaster-risk-reduction frameworks rarely extend beyond emergency dental-trauma response to clinic resilience or continuity of prevention (16, 42). The omission linking oral health and climate adaptation is consistent across national and international oral health policy instruments (2, 15), pointing to systemic health security governance failures.

Three structural factors sustain it. First, dental professionals with public health expertise are seldom represented in the forums where climate-and-health priorities are set, so oral health is omitted by default rather than by decision (43, 44). Second, the absence of standardized, internationally comparable metrics is a practical barrier: we could not identify any oral health indicator among those tracked by the Lancet Countdown (45, 46). Third, and most importantly, these compound into a self-reinforcing cycle. Without expert representation, relevant policies and plans omit oral health. Without measurable oral health considerations of climate impacts, the absence of data justifies continued exclusion (41, 43). The exclusion becomes self-justifying; this circularity, rather than any well-founded judgment that oral health is unimportant, explains its peripheral status and means the remedy lies as much in governance and representation as in new data.

Two further factors deepen the disconnect. Professional education has been slow to incorporate planetary health, so successive cohorts enter clinical practice without the tools to recognize or respond to environmental threats to care (47). And financing reinforces invisibility: as climate priorities are set, oral healthcare risks deprioritization, leaving little capacity to invest in resilience even where the need is acknowledged (48). The result is a workforce under-represented, under-prepared, and under-resourced for the climate change conditions it now faces.

It may be argued that emergency triage rightly privileges life-threatening conditions, and that deferring oral healthcare during a crisis is a defensible allocation of scarce resources. We accept triage at the point of care but reject its extension into planning. Deferral is rational only if care resumes promptly; where clinics, water supply, supply chains, and staffing have not been made resilient, deferral becomes indefinite, allowing disease to progress to more complex and costly interventions, including potentially preventable hospitalizations. Disrupted access and acute needs such as dental trauma are documented in post-disaster settings (16), adding avoidable load to stressed services, and excluding specific health domains from climate action is a recognized pathway to inequitable adaptation (43). Treating oral healthcare as dispensable in planning is a false economy that defers rather than avoids costs, shifting them onto those least able to bear them.

## 4 A framework for integrating oral health into planetary-health governance

Embedding oral health within planetary-health governance requires four mutually reinforcing health system shifts, which create co-benefits for both oral health and environmental sustainability.

First, recognize oral health and care delivery as climate-critical infrastructure. National adaptation plans, health national adaptation plans, and health-system resilience frameworks should explicitly include dental infrastructure, the workforce (including non-dental professionals with oral health competencies), and service continuity alongside hospitals and primary care, drawing on the WHO operational guidance for climate-resilient and low-carbon health systems (49–51). In practice, this means naming oral health in national adaptation planning, expanding the scope of practice of non-dental professionals delivering preventive oral healthcare, and including dentistry in critical-infrastructure audits and resilience standards for cooling, backup power, flood protection, and water security. None of this requires significant budgets for infrastructure investment; oral health and oral healthcare depend on the same systems these frameworks are designed to address.

Second, develop region-specific vulnerability assessments. Adaptation cannot be one-size-fits-all. Supply-chain hardening and protection of mobile dental clinics are priorities where flooding dominates; heat-resistant dental infrastructure design, backup power, and water contingency matter most in heat and drought exposed settings; and cyclone-resistant construction with resilient logistics is paramount for small island states and cyclone-exposed regions. Practical measures range from water-efficient practices and backup instrument sterilization, water storage and rainwater harvesting, and solar power with battery storage, to pre-positioned supplies and referral pathways that maintain urgent care when dental clinics are inaccessible. Standardized but locally calibrated vulnerability assessments would also generate the comparable data whose absence currently justifies exclusion. Candidate indicators, such as days of service interruption per facility per year, the proportion of facilities with reliable water and energy supply, workforce vacancy and turnover rates in hazard-exposed districts, and continuity of community water fluoridation during drought, could be piloted within existing monitoring frameworks to improve oral health visibility.

Third, protect the oral health workforce in climate-exposed regions, while strengthening the role of non-dental professionals in oral disease prevention and oral health promotion. Climate adaptation frameworks treat health workers as a fixed input; an assumption holding only where posts can be filled; yet climate exposure is itself a determinant of where health professionals choose to work. Rural, remote, and island communities already face recruitment and retention difficulties (52); repeated cyclones, floods, heat events, and fire seasons add damaged housing and facilities, disrupted schooling, rising insurance risk, and the prospect of practicing without reliable water or power. Each shock makes a hard-to-fill post harder still, and the result is a reliance on locums, fly-in-fly-out, and volunteer arrangements that deliver episodic care. Care is then focused on treatment rather than prevention, clinical relationships become fragmented, and cultural continuity is least available in the months after a disaster, when need peaks. Climate-prone communities are the first to experience a loss of essential health services and are the last to have them re-established. Workforce retention should therefore be an explicit objective of climate adaptation planning, supported by climate-resilient staff housing, incentives that price in hazard exposure, and local training pathways, including pivotal roles for dental hygienists, dental and oral health therapists, and locally recruited community-based roles (52, 53). Teledentistry, mutual-aid rosters, and innovative service delivery models should complement rather than replace local capacity when service sites are unreachable (54).

Fourth, establish adaptive financing across the healthcare system**.** Dedicated mechanisms are needed to fund climate-resilient oral healthcare, since it risks further deprioritization as climate funding is allocated (48). The economic evidence is strongest for universal upstream measures: community water fluoridation is consistently reported to be cost-saving or cost-effective, whereas evidence for more targeted clinical interventions is mixed and context-dependent (55, 56). Investment should therefore prioritize upstream prevention, resilient infrastructure, and a stable local workforce; these are likely better value than repeated post-disaster responses, largely dependent on conventional service delivery models.

Climate adaptation need not add to the sector's environmental burden; the measures that build resilience also reduce its carbon footprint. Healthcare accounts for roughly 4%–5% of global greenhouse-gas emissions, and oral healthcare contributes a meaningful share through travel, single-use plastics, and energy- and water-intensive procedures (57, 58). Prevention-orientated care lowers demand for carbon-emission-intensive restorative treatment (44, 56). Renewable-powered cooling, efficient water use, and fewer disposables support both adaptation and decarbonization; a locally resident workforce and teledentistry cut the travel impacts that dominate dental carbon footprints in dispersed populations (54, 59), and dietary sugar reduction offers co-benefits for dental caries prevention and planetary health (60). Treating resilience and sustainability as one agenda strengthens the investment case and situates oral health within planetary boundaries (11).

Realizing this framework requires transdisciplinary collaboration across oral health, climate science, and policy taskforces, shared metrics, oral health indicators within instruments such as the Lancet Countdown, and planetary health in curricula (45–47, 61). One Health offers a ready planetary health governance model (54, 62). A focused research program should follow: standardized vulnerability metrics; longitudinal study of how repeated shocks affect service continuity, workforce retention, and outcomes; and an evaluation of low-carbon, climate-resilient models. Professional bodies representing the oral health workforce have a particular role in having a place at the climate-and-health table, since representation precedes the data, financing, and policy action it makes possible. Two limitations should be noted. First, the seven country settings were selected for contrast and co-author expertise rather than by systematic international representation sampling and grey-literature observations. Second, the urgency, magnitude and duration of climate-related impacts on oral health remain unknown. Our action-orientated recommendations are intended to encourage the generation of that evidence.

## 5 Conclusion

Oral health remains marginal within planetary-health governance despite growing evidence of its exposure to climate disruption and its relevance to health security governance. The exclusion of oral health is largely self-perpetuating, sustained by its invisibility and a lack of representation from peak oral health professional bodies. Recognizing oral health and oral healthcare as climate-critical foundations for planetary health involves assessing regional vulnerability, protecting and reforming the workforce, and creating adaptive financing. Promoting oral health and climate-resilient communities can protect at-risk populations through the disruptions ahead while reducing the sector's own ecological footprint. We call on policymakers, professional organizations, educators, and researchers to bring oral health in from the margins of planetary-health governance.

## Data Availability

The original contributions presented in the study are included in the article, further inquiries can be directed to the corresponding author.
